# Cell death induction by the BH3 mimetic GX15-070 in thyroid carcinoma cells

**DOI:** 10.1186/s13046-015-0186-x

**Published:** 2015-07-22

**Authors:** Martina Broecker-Preuss, Jan Viehof, Holger Jastrow, Nina Becher-Boveleth, Dagmar Fuhrer, Klaus Mann

**Affiliations:** Department of Endocrinology and Metabolism, and Division of Laboratory Research, University Hospital Essen, Hufelandstrasse 55, Essen, Germany; Institute of Anatomy, University Hospital Essen, Hufelandstrasse 55, Essen, Germany; Present address: Department of Clinical Chemistry, University Hospital Essen, Hufelandstrasse 55, 45122 Essen, Germany; Present address: Ruhrlandklinik, University Hospital Essen, Tüschener Weg 40, 45239 Essen, Germany; Present address: Clinic of Nuclear Medicine, University Hospital Essen, Hufelandstr. 55, 45122 Essen, Germany; Present address: Center of Endocrinology Alter Hof München, Dienerstr. 12, 80331 Munich, Germany

**Keywords:** Dedifferentiated thyroid carcinoma, Obatoclax, Molecular targeted therapy, Atypical cell death

## Abstract

**Background:**

The evasion of cell death is one of the hallmarks of cancer, contributing to both tumor progression and resistance to therapy. Dedifferentiated and anaplastic thyroid carcinomas that do not take up radioiodine are resistant to conventional anticancer treatments and patients with these tumors are difficult to treat. BH3 mimetics are a new class of drugs that target anti-apoptotic proteins of the BCL-2 family and promote cell death. The purpose of this study was to analyze the molecular effects of the BH3 mimetic GX15-070 on thyroid carcinoma cell lines and to characterize cell death induced by GX15-070.

**Methods:**

A total of 17 cell lines derived from follicular, papillary, and anaplastic thyroid carcinomas were treated with GX15-070. Cell viability was measured with MTT assay while cell cycle phase distribution and subG1 peaks were determined after propidium iodide staining. We assessed cell death via the caspase 3/7 activity, caspase cleavage products, lactate dehydrogenase (LDH) liberation assays, and a LC3 analysis by western blot. Ultrastructural changes were analysed by electron microscopy of GX15-070-treated cells.

**Results:**

After GX15-070 treatment, the number of viable cells was decreased in all cell lines examined, with IC50 values ranging from 48nM to 3.25 μM. We observed biochemical markers of autophagic cell death and necrosis like LC3 conversion and LDH release after the GX15-070 treatment. Electron microscopy revealed several common characteristic ultrastructural changes like swelling of mitochondria, dilatation of rough endoplasmic reticulum, membrane blebbing and formation of vacuoles. GX15-070 treatment induced DNA fragmentation detected by subG1-peak induction and an arrest in G1 phase of the cell cycle. Caspase activation after GX15-070 incubation was detected but had no effect on viability of cells.

**Conclusions:**

With these experiments we demonstrated the efficacy of the BH3 mimetic drug GX15-070 acting against dedifferentiated thyroid carcinoma cells of various histological origins by the induction of cell death. GX15-070-treated cells underwent non-classical cell death with signs of apoptosis, autophagy and necrosis in parallel. GX15-07 and related compounds thus may be a new therapeutic option for dedifferentiated thyroid carcinoma of various histological subtypes.

## Background

Thyroid carcinoma is the most frequently diagnosed malignant tumor of endocrine organs [1, 2]. Most of thyroid carcinomas (approx. 95 to 97 %) are derived from follicular epithelial cells of the thyroid gland [3, 4]. They are grouped according to pathological criteria into well-differentiated papillary (approx. 80 %) and follicular (approx. 10 %) subtypes. Only about 10 % of thyroid carcinomas are initially classified as poorly differentiated or undifferentiated, anaplastic subtypes [3, 4]. While most patients with differentiated papillary thyroid carcinoma (PTC) or follicular thyroid carcinoma (FTC) are successfully treated with surgery and a subsequent radioiodine treatment to destroy residual tumor cells, in a subgroup of these patients the disease recurs even under TSH suppressive therapy [2, 5]. These patients have different outcomes due to the degree of dedifferentiation of their tumors and due to their ability to take up radioiodine [5, 6]. Undifferentiated and anaplastic thyroid carcinomas (ATC) on the other hand have lost the ability to take up radioiodine and are highly aggressive and lethal tumors [7, 8]. Due to the lack of sufficient radioiodine uptake and the aggressive growth pattern, ATCs and dedifferentiated thyroid carcinomas are difficult to treat and have a bad prognosis. In addition to surgery and therapeutic radioiodine treatment, chemotherapy is another therapeutic option for patients with radioiodine-refractory thyroid carcinoma but is less effective with partial response rates of only 25 % or less [9–11].

Cancer cells are characterized by an imbalance between cell division and cell death pathways. This imbalance is caused by an evasion of growth suppressors and sustained proliferative signaling on the one hand and resistance to cell death on the other hand. In addition to further characteristics, it contributes to the “hallmark of cancer cells” [12]. Cell death can be propagated by various pathways that are regulated by different intracellular signaling cascades, i.e. mainly apoptosis, necrosis, necroptosis, and autophagy [Review: 13].

Apoptosis is a form of regulated cell death characterized by the activation of a family of cysteine protease, the caspases [14]. Following caspase activation, characteristic morphological changes are depicted by apoptotic cells like condensation of chromatin and DNA fragmentation, membrane blebbing and disintegration of the cells into small fragments called apoptotic bodies [15, 16]. Apoptosis is mediated by two signalling pathways, which regulate type 1 and type 2 of apoptosis, the extrinsic (type 1) and the intrinsic (type 2) pathway [17]. The intrinsic pathway is triggered by intracellular signals like oxidative stress or damage of DNA. It is regulated by members of the B-cell lymphoma 2 (BCL-2) family of proteins that contains pro- and anti-apoptotic members that are characterized by the presence of sequence motifs termed BCL2 homology domains (BH domains) [18, 19]. The pro-apoptotic proteins BAK and BAX directly execute apoptosis by dimerization and formation of pores in the outer membrane of mitochondria. In turn, cytochrome c and other apoptotic factors are released from the mitochondrial intermembrane space and the executioner caspases 3, 6 and 7 are activated [17]. BAX and BAK proteins are regulated by anti-apoptotic proteins like BCL-2, BCL-xL and MCL-1 which inhibit BAX and BAK by binding. The extrinsic pathway on the other hand is activated after external activation of death receptors on the cell surface (tumor necrosis factor receptor 1, Fas, TRAIL receptor 1 and 2 [20] which in turn activate executioner caspases 3, 6 and 7 [21].

Cell death by necrosis on the other hand is defined by early plasma membrane permeabilisation and cell swelling followed by cell rupture and release of cellular material. In contrast to apoptosis, the cell nuclei in necrosis remain largely intact [Review:^22^]. Biochemically, lysosomal hydroxylases are often released during necrosis and help in the breakdown of the cell [23]. Necrosis originally was defined as an unregulated form of cell death in answer to severe insults of the cell. It now has become clear that necrosis also can occur in a regulated manner which is called necroptosis and is dependent on activation of specific signalling molecules like receptor-interacting protein 1 (RIP1) [22, 24].

Autophagy as another form of cell death describes the process of digesting the cell´s own cellular components Review: [25]. Macroautophagy, which is mostly referred to as autophagy, is a cellular process of degrading and recycling of organelles and cell components [26]. Autophagy is a regulated cellular process that begins with the formation of a phagophore which develops into a double-membrane-encoded vesicle, the autophagosome [27]. The autophagosome contains cellular components that are degraded by acidic hydrolases after fusion with a lysosome, a process called autolysosome formation [28].

Tumor formation is commonly regarded as diminished cell death that occurs in cells carrying genetic alterations that enable their uncontrolled proliferation. Furthermore, success of cytotoxic tumor and radiation therapies depends on the activation of cell death pathways since cytotoxic agents eliminate tumor cells mainly by inducing apoptotic cell death [29, 30]. The expression of the anti-apoptotic proteins BCL-2, BCL-xL, and MCL-1 is upregulated in many cancer types and these proteins, as pro-survival oncogenes, contribute to the uncontrolled proliferation [31]. Furthermore, an important function of members of the BCL-2 family in resistance to classical tumor therapeutics such as chemotherapy has been shown [Review: 32]. Therefore, anti-apoptotic proteins of the BCL-2 family are interesting targets for a therapeutic inhibition to facilitate apoptosis induction in cancer cells.

BH3 mimetics are a new class of low molecular cancer therapeutics which inhibit anti-apoptotic members of the BCL-2 family by mimicking the binding of BH3-only proteins to the hydrophobic groove of anti-apoptotic proteins [33]. In turn, heterodimerization with pro-apoptotic proteins is prevented and pro-apoptotic proteins can assemble to trigger cell death [34]. One of these BH3 mimetic compounds is GX15-070 (Obatoclax) of which Nguyen et al. [35] demonstrated a binding to all anti-apoptotic BCL-2 proteins inhibiting ligation of pro-apoptotic proteins to their hydrophilic groove. GX15-070 showed potent inhibition of a number of cell lines from different tumors but was less effective in normal cells, which makes GX15-070 and related compounds good candidates for anticancer therapeutics [36–40].

Based on the importance of BCL-2 proteins for uncontrolled proliferation and therapy resistance of malignant tumor cells, we studied the effect of the BH3 mimetic GX15-070 on a panel of 17 thyroid carcinoma cell lines. Our aim was to evaluate the suitability of GX15-070 in this cell model since BH3 mimetics are a potential new therapeutic option for dedifferentiated thyroid carcinomas. The effect of GX15-070 on cell proliferation and cell death induction was studied. The type of cell death induced by GX15-070 was characterized by biochemical methods and by transmission electron microscopy. Expression patterns of various proteins of the BCL-2 family were compared with different sensitivities of the cell lines towards GX15-070.

## Methods

### Compounds and antibodies

GX15-070 (Obatoclax) was obtained from Enzo Life Sciences (Farmingdale, NY, USA). It was dissolved in DMSO to 10 mM, stored in aliquots at -20 °C and further diluted in the appropriate medium. Antibodies were from Cell Signaling Technology (Danvers, MA, USA), the pan-caspase inhibitor N-(2-Quinolyl)valyl-aspartyl-(2,6-difluorophenoxy)methyl Ketone (Q-VD-OPh) was purchased from Merck Millipore (Darmstadt, Germany) and staurosporine was from Selleck Chemicals (Houston, TX, USA).

### Cell lines and cell culture

In this study we used cell lines derived from different thyroid cancer subtypes: SW1736, HTh7, HTh74, HTh83, C643, 8305 and 8505C were descended from ATC. BHT101, B-CPAP, K1 and TPC1 originated from PTC. ML1, RO82W, and TT2609 are FTC cell lines. FTC133, FTC236, and FTC238 are derived from a FTC and its metastases from the same patient, with FTC133 originating from the primary tumor, FTC236 from a lymph node metastasis, and FTC238, being derived from a lung metastasis. The HTh7 [41], HTh74 [42], HTh83 [43], C643 [44], and SW1736 [45] cell lines were a gift from Prof. Heldin (Uppsala, Sweden), all other cell lines and Jurkat leukemia cells that were used as controls, were purchased from ATCC (Manassas, Virginia, USA), ECACC (Salisbury, UK) and DSMZ (Braunschweig, Germany). They were grown in their appropriate media supplemented with 10 % fetal bovine serum (FBS; Life Technologies, Paisley, PA, USA) at 37 °C at 5 % CO_2_.

### Viability testing of cells

Depending on the cell line 1 to 5 x 10^4^ cells were seeded into each well of a 96 well plate within their normal growth medium. Medium was replaced after 24 h with medium without FBS containing 0.1 % bovine serum albumin (BSA), and the concentrations of GX15-070, Q-VD-OPh or a combination of GX15-070 and Q-VD-OPh were added as indicated. After 48 h, viable cells were stained with the Cell Titer Aqueous One Solution MTT assay for two to three hours (Promega, Madison, WI, USA) and optical density was measured at 490 nm using an Emax microplate photometer (Molecular Devices, Sunnyvale, CA, USA). Control values without treatment were performed as 18-fold determinations, while all concentrations of GX15-070 and combinations were tested in 8-fold. Each experiment was performed at least three times. Calculation of results and Student’s t-tests were performed using SoftMax® pro software (Molecular Devices, Sunnyvale, CA, USA). IC50 values (drug concentration that caused a 50 % reduction in MTT assay) were calculated with four parameter logistic function dose–response curves using Sigma Plot™ software (Systat, San Jose, CA, USA).

### Determination of lactate dehydrogenase (LDH) release and caspase 3/7 activity measurement

To measure the release of LDH from damaged cells, the CytoTox-ONE homogeneous membrane integrity assay (Promega, Madison, WI, USA) was used. The Apo-ONE homogeneous Caspase-3/7 assay served to measure the activity of caspases 3 and 7 (Promega, Madison, WI, USA). In short, we seeded and grew the cells as described under “viability testing of cells”, with the only difference that black 96 well plates with a clear bottom were used. On day two, medium was replaced by 100 μl medium with 0.1 % BSA containing the indicated GX15-070 concentrations. After the indicated times 50 μl of medium from each well was transferred to a second 96 well plate and equilibrated to 20 °C. According to the instructions of the manufacturer, 50 μl of CytoTox reagent were added followed by incubation at room temperature (RT) in the dark for 10 min. After adding 25 μl of stop solution, fluorescence was determined with excitation and emission wavelengths of 560 nm and 590 nm, respectively. In each experiment, controls without cells and fully lysed cells as maximum LDH release controls were included. By adding 50 μl of Apo-ONE reagent containing a fluorometric substrate, cell lysis reagent, and buffer, we determined caspase 3 and 7 activity in GX15-070-treated cells in the original stimulation plate. Sixty minutes later, after excitation with 499 nm, fluorescence was measured at 521 nm (emission). All values were determined 8-fold and repeated at least two times. We calculated the results and performed Student’s *t*-test using SoftMax® pro software (Molecular Devices). For positive control of apoptosis induction [46], Jurkat cells were harvested by centrifugation, resuspended in medium with 0.1 % BSA, seeded and staurosporine or staurosporine and Q-VD-OPh were added. On day 2, the 96 well plate was centrifugated, 50 μl of supernatant was discarded and ApoONE assay was performed as described.

### Cell cycle analysis

Cells were seeded in six well plates (1 to 5 x 10^5^ cells per well, depending on the cell line) in their appropriate growth media. After 24 h, media were replaced by media without FBS containing 0.1 % BSA and 0.1 μM GX15-070 was added for 24 h. Cells were harvested and fixed in ice-cold 70 % ethanol. After adding RNase A (60 μg/ml) and propidium iodide (25 μg/ml) in PBS, samples were incubated for 20 min at RT in the dark. Samples were measured with a FACS Calibur flow cytometer (Becton Dickinson, San Jose, CA) and cell cycle stages were analyzed using ModFit LT™ Software (Verity Software House, Topsham, ME, USA).

### Cell stimulation and protein extraction

For ELISA and western blot analyses, cells were seeded on cell culture dishes (15 cm diameter) and grown for one to two days until they reached 80 to 85 % confluence. Full medium was replaced with medium containing 0.1 % BSA and cells were maintained in this medium for one hour before adding 0.1 μM GX15-070. For controls, Jurkat cells were centrifugated, resuspended in medium containing 0.1 % BSA and treated with staurosporine or a combination of staurosporine and Q-VD-OPh. After the treatment times indicated, medium was removed and cells were washed with ice-cold PBS. All further steps were performed on ice. Cell lysis was conducted in lysis buffer containing protease and phosphatase inhibitors (Complete protease inhibitor and phosStop phosphatase inhibitor, Roche Applied Science, Mannheim, Germany). The vehicle-stimulated control cells were maintained for 24 h in medium containing BSA before lysis. Lysates were clarified by centrifugation at 10,000 × g for 10 min at 4 °C. Protein concentration was determined with a modified Bradford assay (Bio-Rad Laboratories, Hercules, CA, USA).

### Cleaved caspase ELISA

Semi-quantitative determination of cleaved caspase 3 (Asp175) as a marker of apoptosis induction was performed by using a specific sandwich ELISA for this cleaved protein (Cell Signaling Technologies). In brief, cells were plated, stimulated, and lysed as described above. Diluted cell lysate (100 μl) containing 100 μg of total cell protein were incubated in each of the antibody-coated wells of the plate at 4 °C overnight. All values were determined four-fold and repeated at least once. After washing, detection was performed by use of an antibody specific for the cleaved protein and a horseradish peroxidase-labelled secondary antibody. TMB substrate reaction was stopped after 30 min at RT and absorbance was determined at 450 nm (EMax microplate reader, Molecular Devices). Results were calculated as percentages of the unstimulated controls using SoftMax® pro software (Molecular Devices).

### Western blot analyses

Western blot analyses were performed to analyze the expression of the BCL-2 family protein members and the effects of GX15-070 on LC3 cleavage. Total protein (30 μg) from untreated or vehicle-treated and GX15-070-treated cells (see above) were denatured by boiling in SDS sample buffer for 5 min. Proteins were separated by means of SDS-PAGE on stain-free polyacryl amide gels (Bio-Rad Laboratories) to enable loading control. After electrophoresis, optical densities of stained proteins in each lane were documented and verified with a CCD camera system and the Quantity One® software, respectively (both Bio-Rad Laboratories). When integrated optical densities of proteins in each lane did not differ more than maximal 10 %, proteins were transferred to a nitrocellulose membrane (Bio-Rad Laboratories). After blocking with BSA, the blots were incubated overnight at 4 °C with the appropriate primary antibody (Cell Signaling Technologies) in TBS buffer containing 0.1 % Triton X100. After washing, an appropriate secondary antibody coupled to horseradish peroxidase was added. Detection of bound antigens was performed with an enhanced chemiluminescence detection kit (Amersham ECL Advance, GE Healthcare, Piscataway, NJ, USA). Using a CCD-camera system (Bio-Rad Laboratories), the signal intensities were evaluated.

### Electron microscopy

Cells were plated and treated as described above. Cell culture supernatant was collected together with the scraped cells and centrifuged. The cell pellets were fixed in a solution of 2.5 % glutaraldehyde in 0.1 M sodium cacodylate buffer (pH 7.4; RT) for 90 min followed by washing in sodium cacodylate buffer (3 x 20 min). After osmification with 1 % osmiumtetroxide in cacodylate buffer for 120 min the cells were washed again. Then aqueous ethanol solutions were applied (30 %–50 %–70 %) followed by 70 % ethanol containing 1 % of uranyl acetate for 60 min. After this procedure, 80 %, 90 %, and 96 % ethanol (45 min each) and absolute ethanol (3 x 15 min each) was applied. After this we administered propylenoxide (2 x 20 min) followed by EPON™ solutions in propylenoxide with increasing EPON™ concentrations (propylenoxide : EPON™ = 3:1, 1:1, 1:3; 60 min each) and finally pure EPON™ overnight at RT. For the polymerization a heated storage device (60 °C, two days) was used. After trimming solid EPON™ blocs were cut on a Reichert-Jung Ultracut E®-ultramicrotome, which had been set to a thickness of 60 nm. Sections were then mounted on 200 Mesh hexagonal copper grids and treated for 6 min with 1 % aqueous uranyl acetate solution before exposing them for three minutes to lead citrate (0.4 %) for contrast enhancement. A Zeiss transmission electron microscope (EM902A) was used for investigation at 80KV applying magnifications ranging from 2,500 to 145,000×. Digital image acquisition was performed with a Morada slow-scan-CCD camera connected to a PC running ITEM® 5.0 software (both Olympus Soft-imaging-systems, Münster, Germany).

### Statistical analysis

Statistical analysis was performed by means of the unpaired Student’s *t*-test. P-values <0.05 were considered as statistically significant. For the statistical analysis the Soft Max® pro software (Molecular Devices) and SPSS Statistics (IBM Inc, Armonk, NY, USA) were utilized.

## Results

### GX15-070 decreased viability of thyroid carcinoma cell lines

**Fig. 1:**
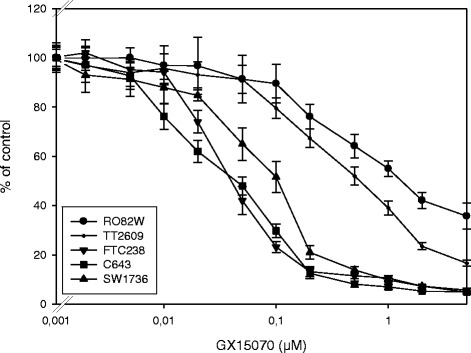
Decreased viability of thyroid carcinoma cells after incubation with GX15-070. Cells were cultured with increasing concentrations of GX15-070 or vehicle control for 48 h and viability was assessed by MTT assay. Values are reported as percent of vehicle control ± standard deviation. Values represent mean values of eight-fold determinations of a representative experiment. IC50 values for all cell lines examined are shown in Table 1

To investigate the effect of a GX15-070 treatment on the viability of thyroid carcinoma cells that had been derived from different histological subtypes, we treated 17 cell lines from anaplastic, papillary, and follicular thyroid carcinoma with GX15-070 or vehicle for 48 h and assessed the percentage of viable cells compared to controls. GX15-070 treatment decreased the number of cells in all 17 thyroid carcinoma cell lines analyzed, although to a variable degree (Table 1). *In vitro* proliferation assay data revealed IC50 values in a wide range of concentration (0.048 to 3.25 μM). The lowest IC50 values of <0.1 μM were found in follicular FTC133, FTC236, and FTC238 cells, papillary BHT101 cells and anaplastic C643 and HTh7 cells. FTC238 and C643 cells were the most sensitive cell lines (IC50 of 0.048 and 0.049 μM; Table 1). High IC50 values (>1 μM) were determined in follicular RO82W cells, papillary K1 cells, and anaplastic 8305 cells. Papillary K1 cells depicted the highest IC50 value of 3.25 μM (Table 1). Follicular ML1 and TT2609 cells, papillary B-CPAP and TPC1 as well as anaplastic SW1736, HTh74, HTh83, and 8505 cells had IC50 values in the medium range between 0.1 and 1.0 μM (Table 1). As examples, results for RO82W, TT2609, FTC238, C643, and SW1736 cells are shown in Fig. 1. Overall, the GX15-070 treatment decreased the quantity of viable cells in all 17 thyroid carcinoma cell lines examined. The IC50 values for GX15-070 treatment ranged between 0.048 and 3.25 μM, but no correlation between histological origin of cell lines and sensitivity towards GX15-070 was obvious. There also was no correlation between the doubling time of cell lines (Table 1) and sensitivity towards GX15-070 since in the group of the fastest growing cells (FTC238, TPC-1 and 8305), two cell lines (FTC238 and TPC-1) had low IC50 values of 0.048 and 0.21 μM, while 8305 cells depict a high IC50 value (2.16 μM; Table 1). Furthermore, ML1 cells which had the longest doubling time of 68 h, showed an IC50 value in the middle range of 0.16 μM (Table 1).

**Table 1:** Cell line origin, doubling times and IC50 values of all thyroid carcinoma cell lines examined after 48 h of GX15-070 treatment (MTT assay), mean of three independent experiments

Cell line	Origin	IC50 GX15070 (μM)	Doubling time (h)
FTC133	FTC	0.052	42
FTC236	FTC	0.089	37
FTC238	FTC	0.048	30
ML1	FTC	0.16	68
TT2609	FTC	0.62	52
RO82W	FTC	1.12	48
BHT101	PTC	0.072	46
B-CPAP	PTC	0.15	36
TPC-1	PTC	0.21	30
K1	PTC	3.25	38
SW1736	ATC	0.11	37
C643	ATC	0.049	31
HTh7	ATC	0.078	36
HTh74	ATC	0.21	43
HTh83	ATC	0.23	32
8305	ATC	2.16	30
8505	ATC	0.16	37

### Expression of BCL-2 family members

**Fig. 2:**
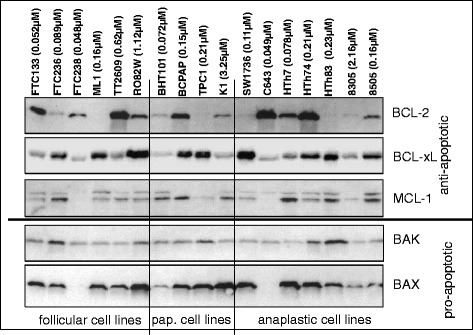
Expression of proteins of the BCL-2-family in various thyroid carcinoma cells. In addition to the cell line denotations, IC50 values for GX15-070 incubation from Table 1 are shown. Equal protein loading was controlled by in-gel protein staining (see “Methods”)

We analyzed protein expression profiles of some BCL-2 family members in all cell lines, since there was no obvious correlation between the sensitivity of the different cell lines against GX15-070 and the thyroid carcinoma subtype from which they had been derived. Results are depicted in Fig. 2 and related to the IC50 values of cells with GX15-070 as shown in Table 1. The cell lines showed variable expression levels of proteins of the BCL-2 family. The expression of the pro-apoptotic protein BAK was relatively consistent in different cell lines with FTC236 and HTh83 showing a slightly higher expression than the other cells (Fig. 2). An expression of BAX was not detectable in sensitive FTC238 cells (follicular origin) and sensitive C643 cells (anaplastic origin). In intermediate BHT101 (papillary) and 8505 (anaplastic) cells as well as in insensitive 8305 cells (anaplastic origin) BAX expression was weak.

BCL-2 expression was the highest in the GX15-070-sensitive cell lines FTC133, C643, and HTh7, the intermediate cell lines TT2609, B-CPAP and HTh74, and in the insensitive cell line RO82W (Fig. 2). No or very weak BCL-2 expression was found in the intermediate cell lines ML1, TPC1, HTh83, and the insensitive cell line 8305 (Fig. 2). BCL-xL expression was strong in the GX15-070 sensitive cell line FTC236, the intermediate cell lines ML1, B-CPAP, TPC1, SW1736, HTh74, HTh83 and 8505 as well as in the insensitive K1 cell line (Fig. 2). The expression of MCL-1 was more consistent among cell lines with the sensitive FTC238 and C643 cells, the intermediate TPC1 cells, and the GX15-070 insensitive cell line 8305 exhibiting the lowest MCL-1 expression (Fig. 2). With respect to the histological origin, there again was no obvious correlation of expression of the anti-apoptotic proteins BCL-2, BCL-xL and MCL-1 with thyroid carcinoma subtype of cell lines (Fig. 2). In summary, we found no correlation between the GX15-070 sensitivity and the expression of single proteins of the BCL-2-family or the expression of pro- and anti-apoptotic BCL-2 family proteins.

### Cell cycle analyses after GX15-070 exposure

**Fig. 3:**
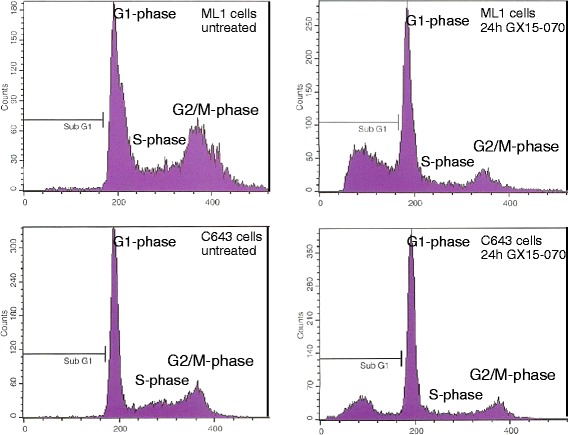
Cell cycle changes in ML1 and C643 cells after incubation with 0.1 μM GX15-070 for 24 h. Cell cycle analysis was conducted using FACS, results are shown as examples. Besides the increase in SubG1 peak, in the remaining living cells an increase in the percentage of cells in G1 phase and a decrease in the percentage of cells in G2/M phase and S phase were observed. Values for all cell lines examined are depicted in Table 2

Cell cycle analyses and the following experiments to determine the kind of cell death caused by GX15-070 were performed in the following six cell lines: FTC236 (follicular), ML1 (follicular), BHT101 (papillary), SW1736 (anaplastic), HTh7 (anaplastic), and C643 (anaplastic). SW1736 anaplastic cells were shown to be derived from a tumor that originated from a papillary thyroid carcinoma due to a *BRAF*^*V600E*^ mutation [47] and all six cell lines chosen had IC50 values around 0.1 μM. Cell cycle analyses after 24 h treatment with 0.1 μM GX15-070 showed a significant increase of cells in the subG1 fraction in all cell lines analyzed, pointing to cell death and DNA fragmentation which were induced by the GX15-070 treatment (Table 2 and Fig. 3). The percentage of cells in subG1 peak was the highest in GX15-070-treated ML1 follicular cells (34.8 %) followed by papillary BHT101 (27.9 %) and anaplastic C643 cells (21.7 %). The lowest value for the percentage of cells in subG1 peak after GX15-070 treatment was achieved in anaplastic HTh7 cells (4.1 %), while follicular FTC236 and anaplastic SW1736 cells exhibited medium values (9.9 % and 9.3 %; Table 2). The remaining living cells from all six cell lines depicted a significant increase in the percentage of cells in the G1-phase of the cell cycle with 75.6 % to 82.2 % of all living cells resting in G1-phase (Table 2). After the GX15-070 treatment, the percentage of cells in the G2/S-phase and the M-phase of the cell cycle was diminished in all cell lines examined (Table 2).

**Table 2:** Distribution of cell cycle phases in vehicle-treated thyroid carcinoma cells and cells treated for 24 h with 0.1 μM GX15-070

Cell line	Stimulation	% subG1	% G1	% G2/M	% S
FTC236 (FTC)	unstimulated	0.3 ± 0.04	36.7 ± 2.4	17.0 ± 1.1	46.3 ± 3.1
	24 h GX15-070	9.9 ± 2.4 *	81.9 ± 5.3 *	12.2 ± 1.7 *	6.0 ± 0.9 *
ML1 (FTC)	unstimulated	1.2 ± 0.3	39.4 ± 1.7	28.5 ± 1.5	32.1 ± 1.8
	24 h GX15-070	34.8 ± 3.5 *	82.2 ± 5.4 *	14.3 ± 0.9 *	3.5 ± 0.4 *
BHT101 (PTC)	unstimulated	3.7 ± 0.6	52.5 ± 3.9	16.3 ± 1.1	31.3 ± 2.2
	24 h GX15-070	27.9 ± 3.0 *	76.1 ± 5.4 *	15.4 ± 1.4	8.6 ± 1.0 *
SW1736 (ATC)	unstimulated	2.4 ± 0.2	35.9 ± 1.7	24.3 ± 2.0	39.8 ± 2.5
	24 h GX15-070	9.3 ± 1.3 *	77.5 ± 5.5 *	11.8 ± 0.9 *	10.8 ± 1.1 *
C643 (ATC)	unstimulated	0.7 ± 0.1	54.5 ± 4.2	13.9 ± 0.9	38.6 ± 2.7
	24 h GX15-070	21.7 ± 2.7 *	75.6 ± 5.8 *	10.4 ± 1.2 *	14.0 ± 0.9 *
HTh7 (ATC)	unstimulated	1.1±0.2	55.5 ± 3.9	11.3 ± 0.9	33.2 ± 1.8
	24 h GX15-070	4.1 ± 0.3 *	80.0 ± 5.7 *	3.9 ± 0.2 *	16.1 ± 1.0 *

Values for G1-, G2/M-, and S-phases are determined for the living cells that were not included in the sub-G1-peak. * indicates significant changes (p < 0.05, Student’s *t*-test)

### Analysis of cell death in GX15-070 treated cells

Cell death analyses were performed in FTC236, ML1, BHT101, SW1736, HTh7, and C643 cells to evaluate if GX15-070-treated thyroid carcinoma cells are growth-inhibited or actively undergo cell death. Furthermore, these analyses should elucidate the kind of cell death in GX15-070-treated thyroid carcinoma cells.

**Fig. 4:**
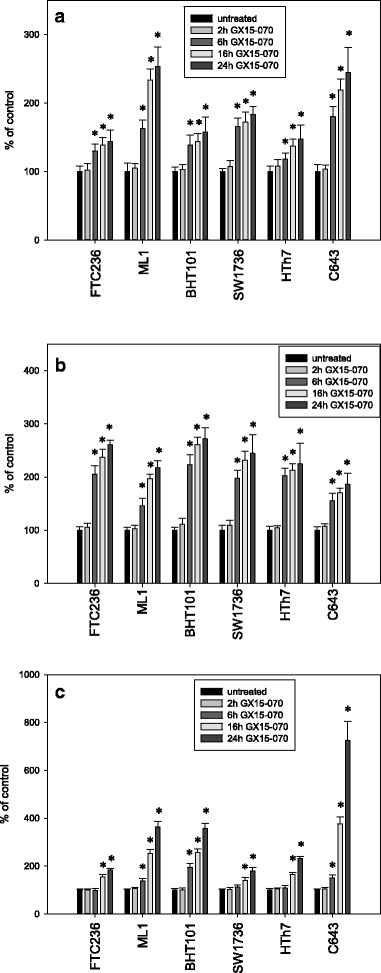
Increased activity of caspase 3 and 7 (**a**), increase in the concentration of cleaved caspase 3 fragments as a sign of progressing caspase activity (**b**) and increased LDH-release into the cell culture medium (**c**) after treatment with 0.1 μM GX15-070 for 2, 6, 16 and 24 h in different thyroid carcinoma cell lines. Values were determined by Apo-One assay, an ELISA specific for caspase 3 fragments and CytoTox-assay and are depicted as percent of vehicle-treated control. Data represent mean values of eight-fold determinations ± standard deviation; * indicates significant increase (p < 0.05, Student’s *t*-test)

Caspase 3 and 7 measurements were performed to investigate possible apoptotic cell death mechanisms after the GX15-070 treatment. As shown in Fig. 4a, caspase activities were significantly elevated after 6, 16 and 24 h of GX15-070 treatment in all six thyroid carcinoma cell lines examined with the highest values after 24 h. ML1 and C643 cells exhibited the highest increase (253 % and 245 % of control after 24 h), while in FTC236, BHT101, SW1736, and HTh7 cells, the increase in caspase 3/7 activities were in the same range (144 to 184 % of untreated control after 24 h; Fig. 4a). Moreover, a specific ELISA analysis in GX15-070-treated cells detected a significant increase in cleaved caspase 3 as a sign of an activated caspase after 6, 16 and 24 h, while after 2 h no significant increase was seen (Fig. 4b). The increase in cleaved caspase 3 after the GX15-070 treatment was of the same magnitude in all six cell lines (146 % to 223 % of control after 6 h, 170 % to 261 % of control after 16 h and 186 % to 272 % of unstimulated control after 24 h; Fig. 4b). LDH activity in supernatants of vehicle-stimulated and GX15-070-treated cells was determined to examine the release of this enzyme that would occur because of a disruption of cell membranes by necrosis or secondarily to other kinds of cell death. While after 2 h no significant increase was seen, after 6 h a small but significant elevation of LDH was depicted only by ML1, BHT101 and C643 cells, while all six cell lines examined depicted a significant elevation of LDH content in medium after 16 and 24 h of incubation with 0.1 μM GX15-070 (Fig. 4c). The late LDH release in all cells after GX15-070 treatment pointed to a delayed cell disruption after the GX15-070 treatment which may be, at least in part, due to secondary necrosis.

**Fig. 5:**
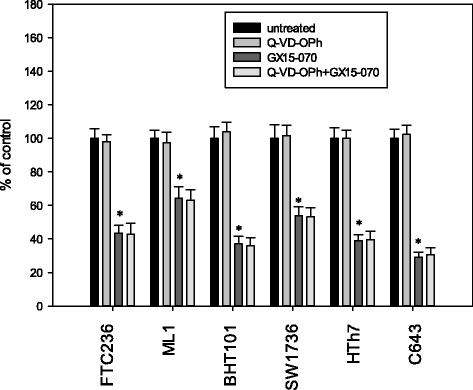
Viability of thyroid carcinoma cells after incubation with 1.0 μM Q-VD-OPh alone, 0.1 μM GX15-070 alone or a combination of both for 48 h. The viability was assessed by MTT assay. Values are reported as percentage of vehicle-treated control ± standard deviation and represent the mean of eight-fold determinations; * indicates significant decrease (p < 0.05, Student’s *t*-test)

Taken together, an increase in caspase activation after GX15-070 treatment together with the DNA fragmentation depicted as SubG1 peak in cell cycle analyses and the delayed LDH release pointed to an activation of the apoptosis machinery in treated cells. To analyze the significance of activated caspases in cell death induction, we co-incubated the cells with 0.1 μM GX15-070 and 1.0 μM Q-VD-OPh, a pan-caspase inhibitor and performed MTT viability assays (Fig. 5). Incubation with GX15-070 alone resulted in the expected decrease in cell viability, while incubation with Q-VD-OPh alone had no significant effect. Combination of both substances depicted the effect of a GX15-070 incubation alone indicating no significant effect of activated caspases on the number of viable cells in the thyroid carcinoma cell lines examined. As a positive control for Q-VD-OPh action, we co-incubated Jurkat cells with 2.5 μM staurosporine which is known to induce apoptosis in these cells [46] and 1.0 μM Q-VD-OPh and measured caspase 3 and 7 activity and cleaved caspase 3 fragments. Caspase 3 and 7 activity was diminished from 1059 ± 178 % (staurosporine alone) to 128 ± 18 % (staurosporine plus Q-VD-OPh) of control, while cleaved caspase 3 fragments were diminished from 1371 ± 176 % (staurosporine) to 141 ± 23 % (staurosporine plus Q-VD-OPh).

**Fig. 6:**
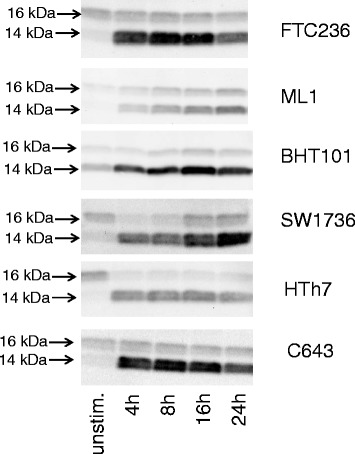
Conversion of LC3-I (16 kDa) to LC3-II (14 kDa) after GX15-070-treatment indicating autophagic processes. Western blot analyses of vehicle-treated and GX15-070-treated thyroid carcinoma cell lines using LC3A/B antibody are shown. Equal protein loading was controlled by in-gel protein staining (see “Methods”)

Furthermore, we used western blot analyses to examine the LC3 conversion as a marker of autophagic cell death in the six cell lines after the GX15-070 treatment. All cells examined in response to the GX15-070 treatment showed a conversion of the 16 KDa LC3-I isoform to the 14 kDa LC3-II isoform within 4 h (Fig. 6). These results point to an involvement of autophagic processes in cell death execution by GX15-070 in thyroid carcinoma cells.

**Fig. 7:**
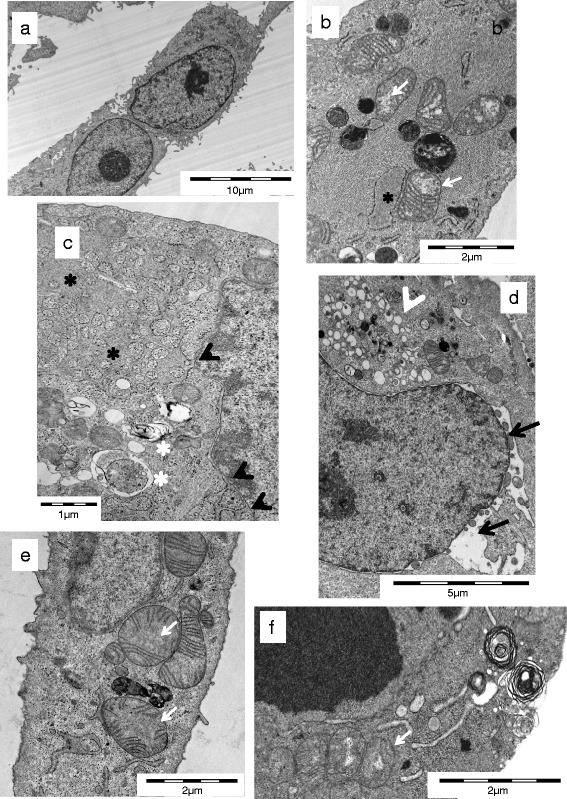
Electron microscopic images of cell death in thyroid carcinoma cells. Untreated SW1736 cells (**a**) with intact membranes, normal organelles, and morphology, (**b**) SW1736, (**c**) HTh7, (**d**) TPC1, (**e**) TPC1, and (f) BHT101 cells treated for 16 (c, d, e) or 24 h (b, f) with 0.1 μM GX15-070 as examples for swelling of mitochondria with loss of cristae (white arrows), blebbing of nuclear membrane (black arrows), extreme dilatation of rER with formation of reticular rER clusters (black asterisks), association of the nuclear membrane with membranes of dilatated rER black arrowhead), vacuoles probably originating from Golgi apparatuses (white arrowheads) as well as lamellar bodies and autophagosomes (white asterisks)

In addition to biochemical analyses of treated cell populations, ultrastructural changes in thyroid carcinoma cells treated with GX15-070 were investigated to see morphological alterations of cell organelles and compartments. Only in a small portion of cells (<1 % of analyzed cells in all cell lines) did we see nuclear changes characteristic for apoptotic cell death. Instead, after 16 and 24 h we noted several other characteristic ultrastructural changes in cells treated with GX15-070: One change was swelling of mitochondria combined with loss of cristae (Fig. 7b, e, f). We observed wide fields of vacuoles probably originating from altered Golgi apparatuses (Fig. 7d) and dilatation of rough endoplasmic reticulum (rER) with formation of reticular rER clusters in cells of all cell lines (Fig. 7b, c) We also noted blebbing and partial destruction of the nuclear membrane in cells of all cell lines analyzed after GX15-070 treatment (Fig. 7d, f). Interestingly, in some cells we observed an association of the nuclear membrane with membranes of extremely dilatated rER (Fig. 7c). Moreover, all cell lines contained cells exhibiting lamellar bodies after GX15-070 treatment (Fig. 7f). After 48 h of GX15-070 treatment, in cells from all cell lines examined, we observed a secondary necrosis with collapse of plasma membrane and organelles and spillage of cell content (not shown). In view of all ultrastructural changes observed we conclude that GX15-070 treatment caused a characteristic damage in thyroid carcinoma cells that was not consistent with any typical form of cell death and which also affected mitochondria and other cell organelles.

## Discussion

In this study we showed the efficacy of the BH3 mimetic drug and pan-BCL-2 antagonist GX15-070 for cell death induction in thyroid carcinoma cells of various histological origins. The dying cells underwent a non-classical way of cell death.

Since the evasion of cell death is one of the characteristics of cancer cells and contributes significantly to tumor progression and resistance to treatment, the pharmacological manipulation of apoptosis-related pathways is a promising strategy for cancer treatment [12]. The resistance to cell death by apoptosis can be mediated through an overexpression of anti-apoptotic proteins of the BCL-2 family, whose expression often correlates with drug resistance [48, 49]. BH3 mimetics are a class of low-molecular weight substances that manipulate the balance of pro- and anti-apoptotic mechanisms by inhibiting BCL-2 proteins. GX15-070 is one of these BH3 mimetic drugs and has already been investigated in some pre-clinical models of various tumors. It has shown antitumor activity e.g. in leukemia, lymphoma, and lung cancer cells [36, 37, 50–52]. Furthermore, GX15-070 has recently shown anti-tumor effects in two mouse cell lines derived from mice with thyroid-specific activation of *Kras* and deletion of *p53*, which develop PTC and subsequently poorly differentiated thyroid carcinoma and ATC and one human ATC cell line [53].

In our experiments, GX15-070 showed inhibitory effects on the numbers of viable cells in all 17 human thyroid carcinoma cell lines examined, although to a variable degree as measured per IC50 value (Fig. 1 and Table 1). The IC50 values for most thyroid carcinoma cell lines (13 of 17) investigated were below 0.50 μM and thus lower than the values that had been seen in most other human cell models (lung cancer cells: 1-2 μM [37]; CLL cells: 1.7 μM at 48 h [54]) which may, at least in part, also be due to different culture conditions and cell culture media. In contrast, Champa et al. [53] described IC50 values in the low nanomolar range for inhibition of two mouse cell lines derived from thyroid tumors. We observed no relationship between the expression of the proteins of the BCL-2 family and the sensitivity against GX15-070. Furthermore, no correlation was found neither between the GX15-070 sensitivity and the thyroid carcinoma subtype from which the cell lines had been derived nor the doubling time of cell lines. With respect to the putative mechanism of the cell death induction after GX15-070 stimulation, an expression of BAK and BAX would be essential for apoptosis induction. This is supported by the work of Nguyen et al. [35], who demonstrated the failure of GX15-070 in apoptosis induction in mouse kidney cells deficient for BAX and BAK. In BAK/BAX double knockout mouse embryonic fibroblast cell lines GX15-070-induced cell death was prevented [55]. Konopleva et al. [52] showed the dependency on BAK and BAX expression for a cell death induced by GX15-070 in AML cells. Furthermore, in cholangiocarcinoma cells, GX15-070 was shown to be a direct activator of BAX [56]. In contrast, in our cells no correlation between expression of these two pro-apoptotic proteins and sensitivity against GX15-070 was obvious: there was no BAX expression and a low BAK expression in FTC238 cells and C643 cells which were both sensitive to GX15-070 (IC50 values of 0.048 μM and 0.049 μM) and a high BAX expression in insensitive RO82W and K1 cells (Fig. 2). These results correspond to those of McCoy et al. [57] who showed that the loss of viability by GX15-070 treatment was not prevented in BAK/BAX knockout mouse embryonic fibroblasts and also to the data of Brem et al. [58] who reported on cell death induction by GX15-070 in lymphoma cells regardless of baseline BAK and BAX levels. The low IC50 values observed in most of our thyroid carcinoma cell lines as well as the lack of correlation of the GX15-070 sensitivity with expression of BAX and BAK and other proteins of the BCL-2 family provided first hints on possible other mechanisms of molecular action of GX15-070 in thyroid carcinoma cells in addition to those of BH3 mimetic activity as also discussed by others [57, 58].

Our biochemical and morphological data in thyroid carcinoma cells treated with GX15-070 support a mixed kind of cell death with biochemical signs of apoptosis and necrosis as well as autophagic cell death. While an increased activity of caspases, which is typical for apoptosis, was seen in all six cell lines examined, the significance of caspase activation for loss of viability in GX15-070-treated thyroid carcinoma cells was low because combined treatment with GX15-070 and the pan-caspase inhibitor Q-VD-OPh did not prevent cell death. These results again fit with those of McCoy et al. [57] who described apoptotic and autophagic cell death induction by GX15-070 in non-small cell lung cancer cells. Furthermore, cell death occurred in conditions with complete inhibition of apoptosis pointing to the relevance of death-inducing pathways other than apoptosis [57]. These authors reported GX15-070 dependent autophagy-induction in parallel with further death inducing mechanisms like vacuolation [57]. Similar findings were also reported by other authors: In leukemia cells, GX15-070 was found to induce both apoptosis and autophagy [59, 60] which in ALL cells are independently regulated [59]. Bonapace et al. reported on GX15-070-induced autophagy-dependent necroptosis in apoptosis-deficient, drug-resistant childhood ALL cells [61]. Moreover, Basit et al. found GX15-070-induced necroptosis and autophagy in RMS rhabdomyosarcoma cells without signs of apoptosis [62]. These authors reported on GX15-070-stimulated assembly of the necrosome on autophagosomal membranes which is interpreted as a connection between GX15-070-stimulated autophagy and necroptosis [62]. Furthermore, GX15-070-induced autophagy may contribute significantly to cell death since GX15-070 treatment was also found to inhibit cathepsin D- and cathepsin L expression which in turn limits the ability of the treated cell to re-use degraded material and thus enables cell death [63]. The results in our cell lines fit very well with these findings. An LDH release typical for cell death by necrosis was found as well in our GX15-070-treated thyroid carcinoma cells. It may be due to necroptosis or secondary necrosis since it was much stronger after 16 and 24 h than after 2 and 6 h. Furthermore, LC3 cleavage and morphological changes indicating autophagic mechanisms were demonstrated in parallel in all thyroid carcinoma cell lines. These findings are in line with literature reports [57, 61, 62]. Cell cycle analyses in addition pointed to DNA fragmentation in GX15-070-treated cells as well as growth inhibition by cell cycle arrest in the G1-phase. These results are in accordance with data from the literature that cell death *in vivo* often is not in accordance with a single cell death mode, but comprises a mix of apoptotic, necrotic and autophagic elements. All kinds of cell death can occur independently of each other or simultaneously resulting in a combined cell death phenotype [64, 65].

Furthermore, electron microscopy revealed ultrastructural signs of atypical cell death with swelling of mitochondria and loss of cristae before cell death as well as disintegration of nuclear membrane, partly extreme dilatation of rER with formation of rER clusters, as well as the formation of vacuoles in the majority of cells, and the occurrence of lamellar bodies and autophagosomes. Electron microscopy revealed necrosis in cells after 48 h of the GX15-070 treatment which corresponds well to the late LDH release depicted through biochemical methods. Similar changes, at least in part, have already described by Vogler et al. [66] and McCoy and coworkers [57]. In non-small cell lung cancer cells, GX15-070 induced a cytoplasmic vacuolation and vesicle formation in the cytoplasm [57]. Vogler et al. [66] described morphological changes in GX15-070 treated CLL cells similar to those seen in thyroid carcinoma cells at least in mitochondria. These authors discuss the death induction of GX15-070 by severe mitochondrial damage. This may compromise the ability of the treated cells to generate ATP by oxidative phosphorylation and in turn impair the ability of the cells to undergo the ATP-dependent process of apoptosis [66]. In our cells, the nuclear membrane was identified as an additional target of GX15-070, maybe involving lamins that were recently identified to be involved in nuclear blebbing [67]. Furthermore, we observed a disintegration of some Golgi apparatuses and generation of partly extremely dilatated rER networks some of which were in conjunction with the nuclear membrane. This proliferation and cluster formation of rER pointed to additional cellular targets perhaps in the context with membrane formation and regulation. These results match those of Albershardt et al. [68] who reported on an induction of the endoplasmic stress response by GX15-070 and other BH3 mimetics. One could speculate that these cellular changes may facilitate death induction by alternative and atypical pathways.

Although GX15-070 was shown to inhibit the binding of a BH3 peptide to recombinant fragments of all anti-apoptotic proteins of the BCL-2 family with Ki values of 1-7 μM [69], our results and those reported by others argue for additional intracellular targets of GX15-070. The low IC50 values for GX15-070 that we observed in most of our thyroid carcinoma cells hint at so far unknown cellular target proteins that have a higher affinity to GX15-070 than BCL-2 proteins. Furthermore, one would expect that BCL-2 antagonists that bind specifically to anti-apoptotic BCL-2 proteins do not lead to a mixed form of cell death but may cause apoptotic cell death.

## Conclusions

GX15-070 was found to efficiently kill dedifferentiated thyroid carcinoma cells although the exact kind of cell death remains to be elucidated. Our findings confirm the results of others [57, 66] that GX15-070 is not specific for proteins of the BCL-2 family and induces a mixed kind of cell death with signs of apoptosis, autophagy and necrosis [57–63, 66]. Moreover, GX15-070 targets additional intracellular structures and organelles like mitochondria and intracellular membranes and induces vacuole formation. GX15-070 and related compounds thus may be valuable as a new therapeutic option for dedifferentiated thyroid cancer, irrespective of the exact target molecules and the kind of cell death it induces.

## Abbreviations

BCL-2: B-cell lymphoma
BH3: BCL-2 homology
PTC: Papillary thyroid carcinoma
FTC: Follicular thyroid carcinoma
ATC: Anaplastic thyroid carcinoma
LDH: Lactate dehydrogenase
BSA: Bovine serum albumin
FBS: Fetal bovine serum
LC3: Microtubule-associated protein 1A/1B-light chain 3
Q-VD-OPh: N-(2-quinolyl)valyl-aspartyl-(2,6-difluorophenoxy)methyl ketone
RIP1: Receptor-interacting protein 1
